# HomeCoRe system for telerehabilitation in individuals at risk of dementia: A usability and user experience study

**DOI:** 10.3389/fmed.2023.1129914

**Published:** 2023-02-17

**Authors:** Sara Bernini, Silvia Panzarasa, Silvana Quaglini, Alfredo Costa, Marta Picascia, Stefano F. Cappa, Chiara Cerami, Cristina Tassorelli, Tomaso Vecchi, Sara Bottiroli

**Affiliations:** ^1^Dementia Research Center, IRCCS Mondino Foundation, Pavia, Italy; ^2^Department of Electrical, Computer and Biomedical Engineering, University of Pavia, Pavia, Italy; ^3^Department of Brain and Behavioral Sciences, University of Pavia, Pavia, Italy; ^4^IUSS Cognitive Neuroscience (ICoN) Center, Scuola Universitaria di Studi Superiori IUSS, Pavia, Italy; ^5^Headache Science and Neurorehabilitation Centre, IRCCS Mondino Foundation, Pavia, Italy; ^6^Giustino Fortunato University, Benevento, Italy

**Keywords:** usability, user experience, neurocognitive disorder, cognitive rehabilitation, telerehabilitation

## Abstract

**Background:**

Telerehabilitation has enabled a broader application of cognitive rehabilitation programs. We have recently developed HomeCoRe, a system for supporting cognitive intervention remotely with the assistance of a family member. The main goal of the present study was to determine usability and user experience of HomeCoRe in individuals at risk of dementia and in their family members. The association between subjects’ technological skills and main outcome measures was evaluated as well.

**Methods:**

Fourteen individuals with subjective cognitive decline (SCD) or mild neurocognitive disorder (mNCD) were recruited to participate in this pilot study. All participants received a touch-screen laptop implemented with the HomeCoRe software. The intervention consisted of 18 sessions and included a patient-tailored adaptive protocol of cognitive exercises. Usability was assessed in terms of treatment adherence and participants’ performance across sessions; user experience *via* self-reported questionnaires and a descriptive diary.

**Results:**

Usability and user experience were overall satisfactory and suggested usability, pleasantness, and high motivation while using HomeCoRe. Technological skills correlated only with the perceived ability to start and/or perform exercises autonomously.

**Discussion:**

These results, although preliminary, suggest that the usability and user experience of HomeCoRe are satisfactory and independent of technological skills. These findings encourage wider and more systematic use of HomeCoRe to overcome the current limitations of in-person cognitive rehabilitation programs and to reach more individuals at risk of dementia.

## Introduction

Mild neurocognitive disorder (mNCD) (1) is defined as a transitional status between normal aging and possible development of early dementia. It is characterized by subjective cognitive complaints and objective cognitive decline greater than expected for individual’s age and education levels, but not interfering with activities of daily life. Subjective cognitive decline (SCD) is a condition referring to the self-perception of worsening cognitive abilities relative to a previous level of performance (2). Existing literature suggests that SCD individuals are at greater risk for dementia than older adults without SCD, as their subjective cognitive decline would in part reflect subtle impairment that has not yet reached the criteria for mNCD diagnosis (3). Given the limited effectiveness of pharmacological treatments in slowing cognitive symptoms in all these “at risk” individuals, cognitive rehabilitation techniques have gained increasing attention in recent years (4–6).

Cognitive rehabilitation can be delivered using traditional paper-and-pencil techniques or by means of more innovative computer-based solutions (7), this thanks to the development of Information and Communication Technologies. Computer-based solutions overcome some limits of traditional approaches, such as time, costs, and individuals’ accessibility, to name a few (8–11). In fact, computerized rehabilitation uses engaging motivational cues and provides real-time feedback; task complexity and response time demands may change frequently during and across sessions, in accordance with individual performance. This allows avoiding over- or under-stimulation and providing more training time in areas of relative weakness. Computer support also saves time for therapists in the preparation of the exercises and allows to record all session parameters for further statistics (12). Telerehabilitation (TR) represents a further development of computer-based rehabilitation, providing assistance to individuals at risk of dementia on a large scale and directly at home (13, 14). In this regard, it is of particular importance that TR tools have a person-centered design, involving final users into the creation, design, and refinement of the software (15). To the best of our knowledge, the available evidence about usability and user experience (UX) associated to cognitive TR in the field of neurodegenerative diseases is still poor and heterogeneous (16–22). For instance, Isernia and colleagues (23) evaluated participants’ experience with iHEAD, which is a telerehabilitation program for both motor and cognitive abilities in Chronic Neurological Diseases (e.g., Parkinson’s disease, multiple sclerosis, and stroke). Jelcic (24) explored the feasibility of a lexical-semantic stimulation *via* TR in early Alzheimer disease.

Usability is defined as the degree to which a particular system can be used with effectiveness, efficiency, and satisfaction by users (25). It can be measured with objective parameters, such as number of completed tasks, time to complete the tasks, number of interventions made by the therapist, and number of errors (26). UX refers to the perceptions, beliefs, emotions, and preferences related to the utilization of the TR system (25). UX can be considered as a subjective dimension assessed by means of validated questionnaires and scales or by non-standardized tools (26). Taken together, usability and UX are used to evaluate the effectiveness of TR system and they should be preliminary assessed (27). A major issue in the field of normal and pathological aging is the lack of familiarity/life experience with advanced technology, which could determine difficulties in the autonomous management of digital devices (28–30). Therefore, it has been stressed the need of accessible and user-friendly TR platforms in aged populations. As consequence, duration and frequency of cognitive rehabilitation sessions need to be adjusted according to participant’s characteristics (31) as well as adherence to treatment and outcome measures must be monitored by the therapist remotely (32).

In the last years, a cognitive rehabilitation (CoRe) software was implemented for an in-person cognitive training (33, 34). CoRe has been shown to be effective in restoring lost brain function and slowing degenerative diseases in early cognitive decline, compared with traditional interventions (35–37). In view of the willingness of treated participants to start/continue CoRe program at distance (38), we have recently developed a “home” version (i.e., HomeCoRe), able to provide a cognitive intervention directly at home (39, 40).

In the present study, we aimed at determining the usability and UX of the HomeCoRe system in individuals at risk of dementia. To this end, we recruited a sample of 14 individuals with SCD or mNCD. Treatment adherence and performances across the treatment sessions were used as usability indicators; UX was evaluated by means of both self-reported quantitative questionnaires and a qualitative session diary exploring personal experience while using the system. In particular, we considered the percentage of completed sessions as primary outcome, while the other usability and UX scores as secondary outcomes. The association between participants’ technological skills and main outcome measures was evaluated as well.

## Methods

### Participants and study design

Fourteen individuals with SCD (*n* = 4) or mNCD (*n* = 10) were recruited from the IRCCS Mondino Foundation of Pavia. This number met the most common sample size requirement for a usability assessment (41, 42). See Table 1 for participants’ characteristics.

**Table 1:** Participants’ characteristics.

	SCD (*n* = 4)		mNCD (*n* = 10)		Total (*n* = 14)	
	Mean	Standard deviation	Mean	Standard deviation	Mean	Standard deviation
Age in years	61.5	6.5	71.0	7.6	68.2	8.4
Gender (% female)	100	-	50	-	65	-
Years of education	12.2	3.7	12.2	4.6	12.2	4.3
CDR	0.0	0.0	0.4	0.1	0.3	0.2
IADL	8.0	0.0	7.8	0.6	7.8	0.5
BDI	16.2	7.1	9.4	6.9	11.4	7.5
SF-36 m	44.7	3.5	41.7	6.2	42.6	5.7
Sf-36 p	41.0	8.4	51.2	2.9	48.3	5.7
CRI-q	106.5	14.7	111.4	16.3	110.0	15.5
TS	2.5	1.0	2.2	1.3	2.3	1.3
MMSE	28.7	0.8	27.0	1.1	27.5	1.3
MoCA	22.0	1.4	21.1	3.3	21.4	2.9
DGS	4.7	0.5	4.5	0.9	4.6	0.8
CBTT	4.7	0.7	4.3	0.5	4.5	0.6
Verbal span	3.8	0.6	41.2	117.3	30.5	99.1
RAVLT-IR	3.2	0.9	1.2	0.9	1.8	1.3
RAVLT-DR	8.8	2.3	4.7	1.9	5.9	2.7
Logical memory	6.8	3.5	4.8	3.2	5.4	3.3
ROCF-delayed recall	19.1	4.8	11.9	5.2	13.9	5.9
RPM	29.8	5.8	30.1	4.6	30.0	4.7
FAB	15.5	1.5	14.6	3.0	14.8	2.6
TMTA	59.0	20.9	94.1	66.7	84.1	58.8
TMTB	65.0	35.5	224.6	198.3	179.0	181.9
Attentive matrices	44.2	8.3	45.3	6.4	45.0	6.6
FAS	31.3	8.6	32.7	10.1	32.3	9.4
SVF	33.0	2.7	34.0	6.1	33.7	5.3
ROCF-copy	34.2	2.0	31.3	4.5	32.1	4.1

SCD, subjective cognitive decline; mNCD, minor neurocognitive disorder; CDR, Clinical Dementia Rating scale; IADL, Instrumental Activities of Daily Living; BDI, Beck Depression Inventory; SF-36 m, Short Form 36 mental sub-dimension; SF-36 p, Short Form 36 physical sub-dimension; CRIq, Cognitive Reserve Index questionnaire; TS, Technological Skills; MMSE, Mini-Mental State Examination; MoCA, Montreal Cognitive Assessment; DGS, Digit Span; CBTT, Corsi’s Block-Tapping Test; RAVLT-IR, Rey Auditory Verbal Learning Test immediate recall; RAVLT-DR, Rey Auditory Verbal Learning Test delayed recall; Logical Memory, logical memory immediate-delayed recall; ROCF, Rey-Osterrieth Complex Figure; RPM, Raven’s Progressive Matrices; FAB, Frontal Assessment Battery; TMTA, Trail Making Test A; TMTB, Trail Making Test B; FAS, phonemic verbal fluency; SVF, semantic verbal fluency.

Inclusion criteria were: (a) age >50 years; (b) education >5 years; (c) a diagnosis of SCD (2) or mNCD (1) based on clinical history, neurological and neuropsychological assessment; (d) clinical dementia rating (CDR) (43) score ≤0.5; and (e) mini-mental state examination (MMSE) (44) adjusted score ≥23.8. Exclusion criteria were the presence of sensory impairments and/or of motor functioning deficits in dominant upper limb. In general, participants were supported in the use of the device and software by a family member. In case they felt particularly independent, the involvement of the family member was not requested.

Individuals once enrolled underwent an in-person baseline assessment concerning clinical questionnaires and neuropsychological measures of about 90 min (T0) using the below-listed tests. Then, they were addressed to the HomeCoRe intervention program consisting in 18 remote at-home sessions (3 sessions/week for 6 weeks, each lasting approximately 45 min/day). Finally, at the end of the 6-week rehabilitation program, participants and family members underwent a final (T1) assessment evaluating usability and UX. We did not collect any neuropsychological assessment at T1, as we were interested in evaluating usability and UX data before carrying out an effectiveness study.

The study was approved by the Institutional Review Board of the San Matteo Hospital, Pavia, and it was carried out in compliance with the Helsinki Declaration. Written consent was obtained from all participants (and possible family members).

### Assessment

See Table 2 for timing and the target user of each evaluation.

**Table 2:** List of measures used in the study at baseline (T0; pre-treatment) and post-treatment (T1; final assessment at the end of the HomeCoRe rehabilitation program).

	T0	T1
**Clinical questionnaires and neuropsychological measures**		
Mini Mental State Examination (MMSE) and Montreal Cognitive Assessment (MoCA)	P	
Digit Span (DGS), Corsi’s Block-Tapping Test (CBTT), Verbal Span, Rey Auditory Verbal Learning Test (RAVLT) Immediate and Delayed Recall, Logical Memory Immediate-Delayed Recall, Rey-Osterrieth Complex Figure (ROCF), Raven’s Progressive Matrices (RPM), Frontal Assessment Battery (FAB), Trail Making Test (TMT) part A and B, attentive matrices, and phonemic (FAS) and semantic (SVF) verbal fluency	P	
Instrumental Activities of Daily Living (IADL)	P	
Beck Depression Inventory (BDI)	P, F	
36-Item Short Form Health Survey questionnaire (SF-36)	P	
Cognitive Reserve Index questionnaire (CRIq)	P	
Self-reported evaluation of Technological Skills (TS)	P,F	
**Usability measures**		
Total number of completed sessions		P
Total number of completed tasks		P
Total time spent for the whole treatment		P
Weighted score		P
**User experience (UX) measures**		
User Experience Questionnaire (UEQ)		P, F
System Usability Scale (SUS)		P, F
Patient Global Impression of Change (PGIC)		P
HomeCoRe User Experience questionnaire (HUXQ)		P, F
Descriptive session diary		P

P denotes measures administered to participants; F measures administered to family members.

**Clinical questionnaires and neuropsychological measures**

- MMSE (44) and Montreal Cognitive Assessment (MoCA) (45) for cognitive screening
- Digit Span (DGS) (46), Corsi’s Block-Tapping Test (CBTT) (46), Verbal Span (46), Rey Auditory Verbal Learning Test (RAVLT) Immediate and Delayed Recall (47), Logical Memory Immediate-Delayed Recall (48), Rey-Osterrieth Complex Figure (ROCF) (49), Raven’s Progressive Matrices (RPM) (47), Frontal Assessment Battery (FAB) (50), Trail Making Test (TMT) part A and B (51), attentive matrices (46), and phonemic (FAS) (47) and semantic (SVF) verbal fluency (48) for neuropsychological evaluation
- Instrumental Activities of Daily Living (IADL) (52) for functional level
- Beck Depression Inventory (BDI) (53) for depressive symptoms
- 36-Item Short Form Health Survey questionnaire (SF-36) (54) for quality of life considering the mental and physical sub-dimensions
- Cognitive Reserve Index questionnaire (CRIq) (55) for cognitive reserve
- Self-reported evaluation of Technological Skills (TS) that participants think they had on a Likert scale with 0 (null), 1 (scarce), 2 (modest), 3 (good), and 4 (excellent) as possible answers.

**Usability**

- Percentage of completed sessions, completed tasks, and time spent to carry out the treatment as measures of adherence
- Overall “Weighted Score” (WS) (33), ranging from 0 to 100 (higher scores correspond to better performances), which is a unique value summarizing participant’s performance (see the “HomeCoRe intervention” section for more information).

**User experience (UX)**

- The User Experience Questionnaire (UEQ) (56) is a measure developed to investigate the subjective impression of users toward the UX of products. Several language versions were constructed and validated, including Italian^1^. It is a semantic differential questionnaire with 26 items consisting of a pair of terms with opposite meanings (e.g.: efficient/inefficient). Participants are asked to rate each item on a 7-point Likert scale from −3 (fully agree with negative term) to +3 (fully agree with positive term). The 26 items are arranged into six scales: Attractiveness (the product should look attractive, enjoyable, friendly, and pleasant, benchmark = 1.04 ± 0.64); Perspicuity (the product should be easy to understand, clear, simple, and easy to learn, benchmark = 0.97 ± 0.62); efficiency (I should perform my tasks with the product fast, efficient, and in a pragmatic way, benchmark = 1.06 ± 0.67); dependability (the interaction with the product should be predictable, secure, and meets my expectations, benchmark = 1.07 ± 0.52); stimulation (using the product should be interesting, exciting, and motivating, benchmark = 0.87 ± 0.62); novelty (the product should be innovative, inventive, and creatively designed, benchmark = 0.61 ± 0.72)
- The System Usability Scale (SUS) (57) is a tool used to quantify the satisfaction of a digital user experience. Scoring instructions of Brooke (57) were considered. The final score ranges from 10 to 100. A cut-off score of 68 indicates a satisfying level of technological system’s usability
- The Patient Global Impression of Change (PGIC) (58) scale is a measure of perceived change in cognitive functioning, autonomy in daily activities, and quality of life after the rehabilitative treatment. Participants respond following the general stem “Compared to how you were before treatment” using the following symmetrical bipolar scale: (1) very much worse, (2) much worse, (3) minimally worse, (4) no change, (5) minimally improved, (6) much improved, and (7) very much improved
- The 8-item HomeCoRe User Experience Questionnaire (HUXQ) (Supplementary Table S1) is a measure created *ad-hoc* referring to other TR tools (23) to investigate specific issues experienced by participants and family members during the HomeCoRe program. Each item was scored on a 5-point Likert scale (0 = never, 4 = always). The questionnaire explores the following domains: Motivation (4 items, Cronbach’s alpha = 0.73); Autonomy in the use of the device (2 items, Cronbach’s alpha = 0.95); Inclusion in the routine (1 item); Technical problems (1 item). For each domain, the mean score is calculated. Given that negatively keyed items (item 3, 4, 5, 6, 7, and 8) have been reverted, higher scores reflect higher levels of user experience for that domain
- Participants were asked to fill in a descriptive session diary, to further gather the subjective impressions about HomeCoRe. In particular, they were stimulated to report any possible difficulty they experienced during each session.

### HomeCoRe intervention

HomeCoRe is a research software tool developed within a long-lasting collaboration between clinicians and bioengineers. At the moment, the tool is limited to Italian speaking participants. The tool allows a patient-tailored intervention aimed to stimulate several cognitive abilities (e.g., logical-executive functions, attention/processing-speed, working memory, and episodic memory) through several sessions of cognitive exercises (see Table 3 for details). It is time-saving for the therapist, as it is ready to use and does not require a continuous manual setting of exercises for each training session. This is because once the therapist remotely set up the treatment plan according to the participant’s cognitive profile (SCD or mNCD), the exercises are carried out in adaptive mode in all sessions. The treatment plan has a weekly structure, so it is repeated for 6 weeks. Each exercise could be carried out several times in each session, depending on the established plan and the level of difficulty achieved. Exercises are presented randomly and their duration could vary from 60 s to about 8 min, depending on the level of difficulty. In particular, during the dynamic generation of exercises, individual performance data are analyzed in order to set the appropriate difficulty level. Participant’s performance data refer to the response accuracy normalized according to the number of aids that he/she has required to solve the task. For each exercise and each level, thresholds are defined to allow difficulty levels to progressively increase in order to stimulate neural plasticity (4, 59, 60). Hence, the system calculates an “overall weighted score” (WS), taking into account the correctness of the answers, the execution time, and the difficulty of the exercises. The score has a value ranging from 0 to 100 and is calculated with this formula: WS = 25*P_type_+25*P_lev_ + 25*P_time_+25*P_resp_.

**Table 3:** Description of HomeCoRe tasks.

Tasks	Description	Main involved skills
Learning of couples	Pairs of words are shown on the screen, the patient must rewrite the second word of the couple when it is shown in a different order.	Long-term memory abilities; learning and re-enactment strategies; visual imagery.
Word categorization	Words belonging to different categories are presented on the screen, the patient must rewrite them in any order but respecting the corresponding category.	Long-term memory abilities; learning and re-enactment strategies; visual imagery; categorical thinking.
Puzzle	The patient must recompose the tiles to form a figure, the whole figure in the simplest levels is shown at the beginning of the exercise.	Visuospatial long-term memory; visual imagery; mental representation and pianification.
Span backward	The patient must write the numbers in reverse order compared to how they were previously heard.	Verbal working memory; processing-speed.
Memory	Tiles that form pairs are shown on the screen, the tiles are turned and the patient has to choose two cards at a time to form all the pairs.	Long-term memory abilities; visuospatial abilities.
Visuospatial matrices	The patient has to store and represent in the correct order on the grid the spatial instructions received (for example up, down, left, right, etc.).	Working memory; visuospatial abilities; processing-speed.
Logical sequences	A sequence of images is shown, the patient must select, among several options, the one that completes the series.	Non-verbal reasoning; mental problem solving; decision making.
Image and sound	An image (small or big) is displayed and a sound (with low or high volume) is played; the patient must evaluate whether size and volume match.	Inhibitory control; processing-speed; working memory.
Unscramble the sentence	Scrambled words are displayed; the patient must select them in the right order to compose a sensible sentence.	Mental and verbal planning; conceptual abstraction abilities.
Unscramble the images	The patient must put the scrambled images in the right order to form a short story.	Planning of activities: problem solving; temporal sequencing; visual attention.
Find the elements	A matrix of random elements (letters or numbers) is displayed, the patient must identify and select all the requested ones.	Sustained and selective attention; visuospatial scanning; processing-speed.

P_type_,P_lev_,P_time_, and P_resp_ are specific scores (from 0 to 1) referring to:

- P_type_ = measures the complexity of the exercise calculated on the basis of the performance of healthy volunteers
- P_lev_ = DL/nDL
- P_time_ = (TT-TR)/TT
- P_resp_ = ACC.

Each of the variables reported in the formulas above has the following meaning:

- DL = difficulty level of the exercise
- nDL = maximum number of difficulty levels foreseen by the exercise
- TT = total time available to perform the exercise
- TR = response time
- ACC = accuracy ranging from 0 (wrong answer) to 1 (completely correct answer).

**Figure 1:**
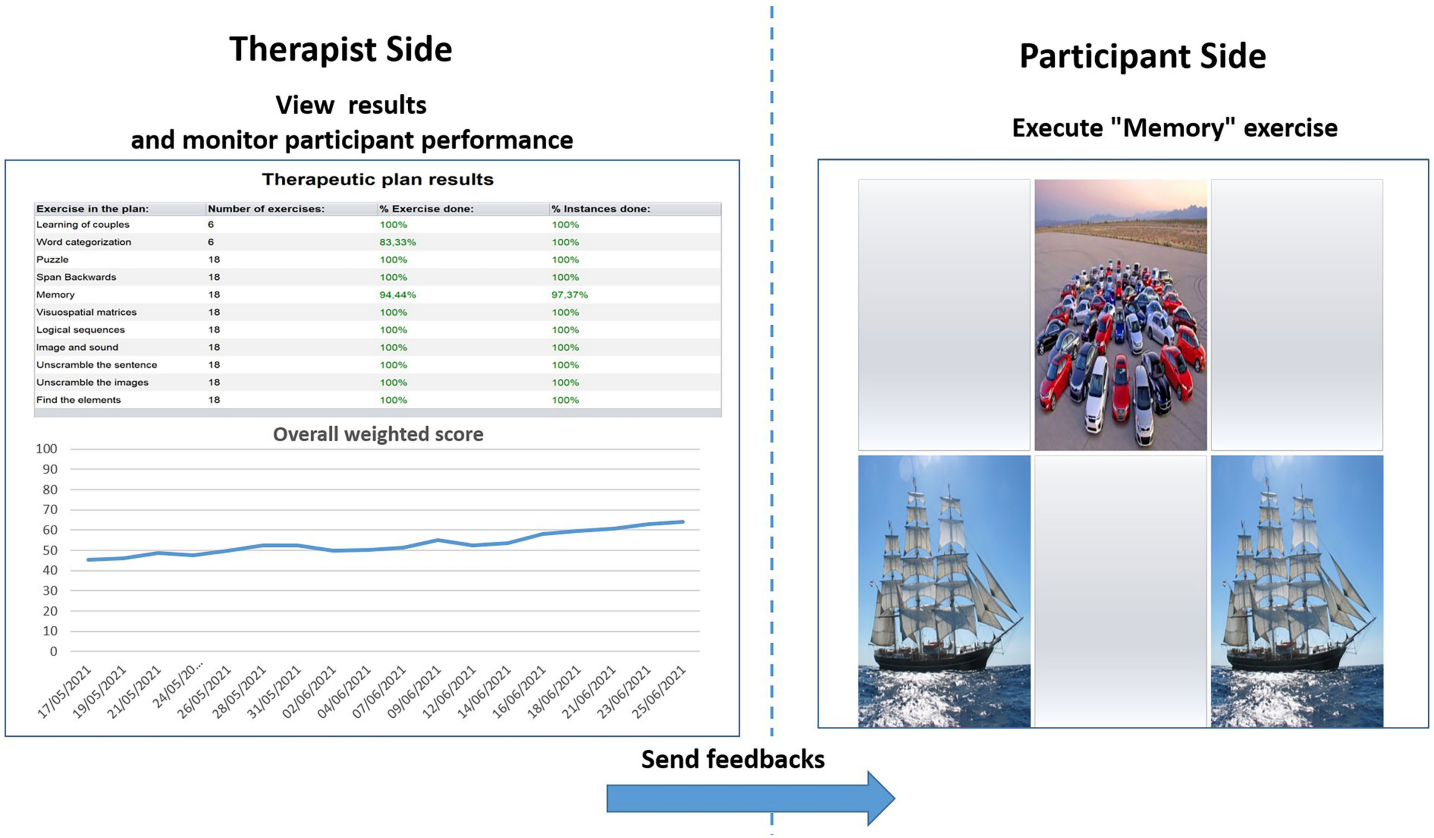
Therapist interface for monitoring performances in terms of weighted score (left) and interface of the participant for the execution of exercises (right). % Exercise done = the amount of exercises carried out until that moment; % Instance done = the number of times that a specific exercise has been performed as planned until that moment; the arrow with “send feedbacks” suggests that the difficulty level for the to-be-performed exercises (right) derives from the trend of treatment performance seen by the therapist (left).

The WS informs the therapist about each participant’s performance in a single value. Hence, WS represents a useful and advantageous index that can be used to assess both the overall outcome of a training session and the global trend of the rehabilitation (see Figure 1).

### HomeCoRe software architecture

HomeCoRe is installed on a touch-screen laptop (password protected and encrypted) that is supplied to the participant by the therapist. Before the beginning of HomeCoRe treatment, the participant and the family member have been trained together at the hospital on the use of the rehabilitation tool at home. This is in order to account for possible differences in baseline technological skills. Then, during the training sessions, participant (with the possible support of his/her family member) goes through each exercise of the treatment until he/she feels familiar with the use of the device. During the rehabilitative program, remote technical support is available when requested. To this aim, the participant is provided with the support team contacts as well as a specific text-messaging section is included in the interface. The treatment sessions can be paused in case of fatigue and resumed at a later time.

**Figure 2:**
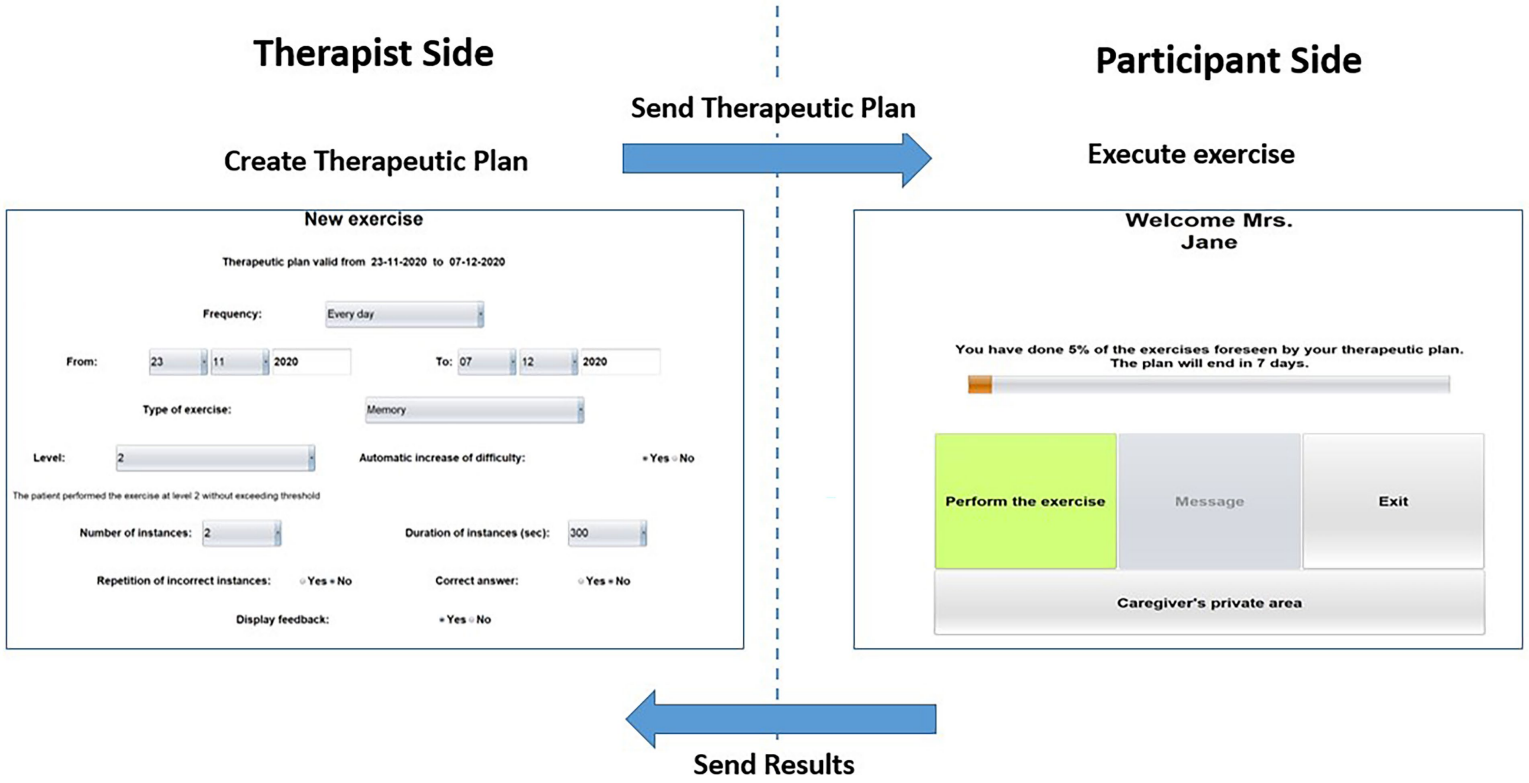
Home page of the therapist side of the interface for setting the requirements for the exercises plan (left) and home page of the participant side of the interface for HomeCoRe (right) in the offline and online modality.

HomeCoRe architecture includes two main components, namely, therapist side and participant side, and a communication system (HomeCoRe Server). The therapist-side dashboard allows to remotely set and monitor all parameters of the treatment plan (e.g., frequency and duration of the plan, type of exercises, difficulty level). The interface of the participant is simple and it allows to view/execute the exercises of the day and to send the results to the therapist (see Figure 2).

The HomeCoRe system can be used online or offline, in the case that the Internet connection of the participant is not available. In the online mode, the communication between therapist side and participant side takes place automatically through a dedicated communication protocol managed by the HomeCoRe Server, while in the offline modality, some manual operations are required for loading the therapeutic plan and save results report on an external memory support (e.g., USB key or hard disk). The communication with the therapist is asynchronous.

### Statistical analysis

Statistical analysis on quantitative outcome measures were conducted using SPSS software. Spearman’s correlations were used for evaluating relationships between participant’s technological skills and usability and UX measures. Wilcoxon signed-rank test was used for comparing WS scores across the intervention (session 18 vs. session 1). A *p* ≤ 0.05, corrected for multiple comparisons if appropriate, was considered as significant.

We used a qualitative content analysis (61, 62) to evaluate subjective impressions recorded on session diary. Meaning units were identified and then condensed into themes (62). Revision of themes occurred until saturation was reached. A second coder reviewed coding and theme development for agreement.

## Results

### Participants’ characteristics

Fourteen participants were enrolled in the study. They all successfully completed the 6-week intervention program. Eleven family members were involved in the study. Three individuals were without a family member because they did not need it, being very independent in the use of the device and software. The mean technological skills (range 0–4) was 2.1 ± 1.2 for participants and 2.4 ± 0.8 for family members.

### Usability

Participants completed at least the 96.1 ± 7.3% of the scheduled sessions and the 94.5 ± 5.5% of the scheduled exercises (see Table 4). They spent 14.2 ± 3.7 h carrying out the 6-week treatment. Participants improved their overall WS performance at the HomeCoRe tasks at the end of the treatment (session 18 vs. session 1; *Z* = −3.11, *p* = 0.002) reporting an average WS of 50.4 ± 3.5 at session 1 and of 59.2 ± 6.0 in the last session of the treatment.

**Table 4:** Means and standard deviations for usability indices (left panel) and correlations with participants’ Technological Skills (right panel).

	Usability indices		Correlation with Technological Skills	
	Mean	SD	*r*	*p*
**Treatment adherence**
Completed sessions (%)	96.1	7.3	0.27	0.34
Completed tasks (%)	94.5	5.5	0.44	0.12
Time spent for the treatment (hours)	14.2	3.7	−0.06	0.85
**Weighted Scores**
Session 1	50.4	3.5	0.06	0.84
Session 18	59.2	6.0	0.25	0.38

### User experience

#### Participants

Participant’ UX as resulted from self-reported questionnaires is reported in Table 5.

**Table 5:** User Experience assessed *via* self-reported questionnaires (means and standard deviations) for participants (left panel) and family members (right panel).

	Participants				Family members	
	Self-reported questionnaires		Correlation with Technological Skills		Self-reported questionnaires	
	Mean	SD	*r*	*p*	Mean	SD
**UEQ**
Attractiveness	3.4	1.1	0.30	0.30	3.2	1.2
Perspicuity	3.1	1.6	0.07	0.80	3.0	1.2
Efficiency	3.6	1.3	0.06	0.84	3.1	1.7
Dependability	2.8	1.2	0.12	0.68	3.0	1.5
Stimulation	3.6	0.9	0.25	0.39	3.2	1.3
Novelty	3.5	1.4	0.36	0.20	3.3	1.7
**SUS**	86.1	18.3	0.54	0.051	88.2	12.3
**PGIC**	3.9	1.0	0.22	0.44		
**HUXQ**
Motivation	3.1	0.6	0.41	0.15	2.6	0.7
Autonomy	3.1	1.4	0.71	0.004	2.8	1.5
Inclusion	4.0	0.0	-	-	2.5	0.8
Technical problems	3.5	0.6	−0.19	0.52	2.5	1.1

For participants is also reported the correlation with their Technological Skills. UEQ, User Experience Questionnaire; SUS, System Usability Scale; PGIC, Patient Global Impression of Change; HUXQ, HomeCoRe User Experience Questionnaire. *p < 0.05.

In all UEQ subscales, we obtained mean values corresponding to the benchmark interval for the excellent category. It means that participants had a good impression of both classical usability (efficiency = 3.6 ± 1.3; perspicuity = 3.1 ± 1.6; dependability = 2.8 ± 1.2) and user experience (attractiveness = 3.4 ± 1.1; novelty = 3.5 ± 1.4; stimulation = 3.6 ± 0.9) aspects.

As for the SUS (range 0–100), the mean total score was 86.1 ± 18.3, above the benchmark. Hence, participants perceived a good usability of HomeCoRe.

For the PGCI (range 1–7), participants’ answers ranged between 3 and 6. The mean total score was 3.9 ± 1.0. Overall, individuals perceived an improvement in their cognitive status after the intervention with HomeCoRe.

For the HUXQ (range 0–4 for each domain), all domains obtained a mean score above 3, which means that participants reported to be highly motivated and autonomous in the use of HomeCoRe and in its inclusion in their daily routine, as well as they did not report particular technical problems.

For what concerns the treatment diary, we found 3 themes related to HomeCoRe criticisms: (1) study time for a specific task, i.e., time to process the stimuli presented by an exercise; (2) execution time, i.e., time for providing answers; and (3) task difficulty. Almost half of tasks did not present criticisms: *Word categorization*, *Memory, Logical sequences*, *Unscramble the sentence*, and *Find the elements*. By contrast, *Learning of couples* and *Span backward* resulted as particularly difficult for 64% (*n* = 9) of participants; whereas *Puzzle* and *Image and sound* were challenging for 29% (*n* = 4) of participants. Twenty-nine percent (*n* = 4) of participants reported concerns in terms of execution time for *Unscramble the images*, and of study time for *Span backward* and *Visuospatial matrices*.

#### Family members

For the UEQ, in all six scales we had mean values corresponding to the benchmark interval for the excellent category in both classical usability (efficiency = 3.1 ± 1.7; perspicuity = 3.0 ± 1.2; dependability = 3.0 ± 1.5) and user experience (attractiveness = 3.2 ± 1.2; novelty = 3.3 ± 1.7; stimulation = 3.2 ± 1.3) aspects.

As the SUS, all family members reported a final score 88.2 ± 12.3 which means that HomeCoRe usability was considered satisfactory.

For the HUXQ, all domains obtained a mean score above 2.5, which reflects that family members perceived a good level of motivation in their relatives in the use of HomeCoRe, as well as they did not report particular difficulties in including the treatment in their daily habits.

### Correlation between technological skills and usability and UX measures

Technological skills correlated significantly only with HUXQ Autonomy domain (*p* < 0.05). We also found a tendency to significance for the correlation between technological skills and SUS scores (*p* = 0.051). No other significant correlations resulted between the score of technological skills and the indices of Usability and UX measures.

## Discussion

TR represents a unique opportunity to guarantee constancy and continuity to cognitive rehabilitation at distance. Therefore, the proper development of TR programs and software in neurodegenerative diseases should result from an integrated view of their usability and UX. Unfortunately, in this field, these issues are poorly considered and rarely assessed (16–22). In the present pilot study, we aimed at measuring usability and UX of a cognitive TR intervention in individuals at risk of dementia. Preliminary assessment of their technological skills was performed in order to evaluate its correlation with usability and UX scores. This is because aging may be associated to difficulties in managing technological devices autonomously (28–30).

Interestingly, when considering the objective measurement of usability, including treatment adherence and WS values, we found a generalized good compliance to HomeCoRe coupled with a satisfactory efficiency of this system, which was not related to participants’ level of technological skills. This result may stem from the fact that HomeCoRe was developed by clinicians based on the characteristics of individuals at risk of dementia (not healthy users) to provide a tool to be used in clinical practice. We also found that the total time for the treatment was about 14 h, which means that participants spent about 45 min per session. This result is in line with our previous experience with CoRe (35–37) in which we highlighted a very good adherence to the treatment when administered in hospital setting, where the dropouts were due to participants’ medical conditions and not to the treatment itself. The adherence rate is a crucial issue for the home-based rehabilitation protocols (63). Individuals with neurodegenerative disorders that could benefit from rehabilitation often do not adhere to a prescribed protocol once it is home-based due to the lack of familiarization with technology and computers (64–66) and to the loss of human interaction (67). The distinctiveness of TR systems such as HomeCoRe, by providing objective measures of the progress of the intervention and allowing remote monitoring and contact with the therapist, could be helpful in overcoming these limitations.

The UX was overall positive for both participants and family members. As regards participants, they expressed a good impression and satisfaction toward the product (data from the UEQ) and perceived usability of the HomeCoRe system (data from the SUS), which was also confirmed by their family members. In addition, feedbacks from the HUXQ suggested that participants considered HomeCoRe as particularly useful and enjoyable, were highly motivated, included the system in their routine, and were able to perform TR activities in autonomy without finding technical problems. From the family member side, some slight differences emerged at the HUXQ with respect to their relatives’ answers. In particular, family members reported more technical problems while using HomeCoRe. However, they agreed in perceiving their relatives as motivated and autonomous and had no difficulty in including the treatment in their daily routines. Even if our UX data could be impacted by the risk of courtesy bias, we believe that the integration of these findings with those concerning the objective measurement of usability could support HomeCoRe as a satisfactory tool and then encourage its use for TR.

Correlation analysis highlighted a significant association between technological skills and the fact of being autonomous in performing HomeCoRe activities. This finding highlights the important role of the family member in supporting those individuals less skilled with technologies. In the future, it could be interesting to evaluate perceived technological skills across the intervention sessions in order to understand if they change while participants become more confident with the system.

Finally, we found that all participants reported a good perception of cognitive changes immediately after HomeCoRe intervention, as resulted from the PGIC, regardless of their technological skills. For what concerns the session diary, participants reported issues in performing some tasks and in the corresponding difficulty levels. Concerning this specific issue, it should be noticed that tasks duration and difficulty level were set as the same duration and level we generally used in the clinical setting (35–37). Consequently, it suggests that some little adjustments (e.g., extra time for training and performing some exercises) could be relevant in the TR session due to the lack of therapist feedbacks generally provided during in-person treatment. In addition, future studies exploring also the point of view of health care figures while using HomeCoRe may provide further insight into their experience. Despite the small sample size, the present results suggest that HomeCoRe could represent a useful and acceptable TR system for individuals at risk of dementia. This is important given the crucial role that technologies will play in future neurorehabilitation models. The availability of effective and feasible TR modalities is indeed critical to address the paucity of healthcare personnel dedicated to cognitive rehabilitation within the neuropsychology services, thus allowing to increase the offer to a wider number of participants. It should be noted that we enrolled participants aged above 50 years. This is because, in some cases, dementia-related impairment manifests prior to older adulthood and such a situation may underline different pathological processes (68, 69). The next step will consist in performing a RCT to explore the effectiveness of HomeCoRe in a broader context of neurocognitive disorders.

This study has some limitations. While the sample size is adequate for a usability study (41, 42), it is too small to draw definitive conclusions about the association of technological skills with outcome measures. Participants with poor familiarity with technological devices and without a compliant family member could be excluded by the use of TR, representing a selection bias for this kind of intervention (70). However, there is evidence about the possibility of using telemedicine devices in individuals with early cognitive impairment living alone, given that the compliance is strictly related to the level of monitoring remotely received (71). Our sample consisted of individuals with SCD and MCI who may have had memory problems that impaired their ability to provide a report about something that happened in the past (i.e., user experience indices). In this regard, it should be noted that we integrated UX measures with those provided directly by the system (i.e., usability indices) that could not suffer from this issue. Finally, our intervention lasted for 6 weeks. There are some evidences (66, 72, 73) showing that the level of adherence and compliance drop significantly after the first 3 months of TR. In any case, we believe that such a length of the intervention could be useful in order to avoid to stress participants and family members. In addition, it is important to consider the HomeCoRe architecture allows to customize and extend the treatment duration according to participants’ characteristics.

## Data availability statement

The datasets presented in this study can be found in online repositories. The names of the repository/repositories and accession number(s) can be found at: Zenodo; https://doi.org/10.5281/zenodo.7595576.

## Ethics statement

The studies involving human participants were reviewed and approved by San Matteo Hospital, Pavia. The patients/participants provided their written informed consent to participate in this study.

## Author contributions

SBe and SBo: study concept and design, acquisition of data, analysis and interpretation of data, and drafting of the manuscript. SP and SQ: analysis and interpretation of data. AC, MP, and CC: participants’ recruitment and data interpretation. SC, TV, and CT: critical revision of the manuscript for important intellectual content. All authors contributed to the article and approved the submitted version.

## Funding

This work was supported by a grant from the Italian Ministry of Health (Ricerca Corrente 2020).

## Conflict of interest

The authors declare that the research was conducted in the absence of any commercial or financial relationships that could be construed as a potential conflict of interest.

## Publisher’s note

All claims expressed in this article are solely those of the authors and do not necessarily represent those of their affiliated organizations, or those of the publisher, the editors and the reviewers. Any product that may be evaluated in this article, or claim that may be made by its manufacturer, is not guaranteed or endorsed by the publisher.

## Footnotes

^1^ www.ueq-online.org
