# Supplementary material for: HomeCoRe system for telerehabilitation in individuals at risk of dementia: A usability and user experience study
Source: Front Med (Lausanne). 2023 Feb 17;10:1129914. doi: 10.3389/fmed.2023.1129914 (PMC9983032; doi:10.3389/fmed.2023.1129914)
Supplement: Supplementary file 1 [file Table_1.pdf]

Supplementary Material

HomeCoRe system for telerehabilitation in individuals at risk of dementia: A Usability and User Experience study

Sara Bernini[1], Silvia Panzarasa[2], Silvana Quaglini[2], Alfredo Costa[3,1], Marta Picascia[1], Stefano F Cappa[4,1], Chiara Cerami[4,1], Cristina Tassorelli[3,5], Tomaso Vecchi[3,1], Sara Bottiroli[6,5*]

*** Correspondence:** sara.bottiroli@mondino.it

Supplementary Table 1. HomeCoRe User Experience Questionnaire (HUXQ)

Questions		Domain	Answers and corresponding scores				
**Participant**	**Family member**		**Never**	**Sometimes**	**Moderately**	**Frequently**	**Always**
1. Do you think HomeCoRe treatment was useful?	1. Do you think HomeCoRe was helpful to your relative?	Motivation	0	1	2	3	4
2. Do you think HomeCoRe treatment was enjoyable?	2. Do you think HomeCoRe was enjoyable for your relative?	Motivation	0	1	2	3	4
3. Have you needed someone to remind you to do HomeCoRe exercises? For example, have you ever needed to be reminded to do HomeCoRe exercises? (R)	3. Was it necessary to encourage your relative to do the HomeCoRe exercises? For example, did you ever have to remind him/her to do the exercises? (R)	Motivation	0	1	2	3	4
4. Have you ever found yourself not doing HomeCoRe exercises	4. Have you ever had to remind your relative to do exercises because	Motivation	0	1	2	3	4

because you did not feel like doing them? (R)	he/she did not feel like doing them? (R)						
5. Have you needed someone to help you start HomeCoRe system? (R)	5. Have you had to help your relative to start the HomeCoRe system? (R)	Autonomy in the use of the device	0	1	2	3	4
6. Have you needed someone to help you do HomeCoRe exercises? (R)	6. Have you had to help your relative to do the HomeCoRe exercises? (R)	Autonomy in the use of the device	0	1	2	3	4
7. Have you had to change your daily habits to use the HomeCoRe system? For example, have you had to change your meal times or give up your afternoon nap? (R)	7. Have you had to change your daily habits in order to allow your relative to use the HomeCoRe system? For example, have you had to change meal times or give up your afternoon nap? (R)	Inclusion in the routine	0	1	2	3	4
8. Have you ever had technical problems while using HomeCoRe system? For example, your computer did not work or you had line problems? (R)	8. Have you ever had technical problems while using HomeCore system? For example, did your computer not work or did you have line problems? (R)	Technical problems	0	1	2	3	4

Note. (R) denotes items with a reverse scoring.
